# Herbal formula Yangyinjiedu induces lung cancer cell apoptosis via activation of early growth response 1

**DOI:** 10.1111/jcmm.14501

**Published:** 2019-06-25

**Authors:** Wenxiao Yang, Yani Kang, Qiang Zhao, Ling Bi, Lijing Jiao, Yunzhao Gu, Jun Lu, Jialin Yao, Di Zhou, Jielin Sun, Xiaodong Zhao, Ling Xu

**Affiliations:** ^1^ Department of Oncology, Yueyang Hospital of Integrated Traditional Chinese and Western Medicine Shanghai University of Traditional Chinese Medicine Shanghai China; ^2^ School of Biomedical Engineering Shanghai Jiao Tong University Shanghai China; ^3^ Institute of Clinical Immunology, Yueyang Hospital of Integrated Traditional Chinese and Western Medicine Shanghai University of Traditional Chinese Medicine Shanghai China; ^4^ Department of Pulmonary, Shanghai Chest Hospital Shanghai Jiao Tong University Shanghai China; ^5^ Shanghai Center for Systems Biomedicine Shanghai Jiao Tong University Shanghai China; ^6^ Cancer Institute of Traditional Chinese Medicine Shanghai University of Traditional Chinese Medicine Shanghai China

**Keywords:** ChIP‐seq, EGR1, lung cancer, traditional Chinese medicine, transcriptome

## Abstract

Traditional Chinese Medicine (TCM) has been extensively used in clinical practices and proven to be effective against cancer. However, the underlying mechanisms remain to be investigated. In this study, we examined the anticancer activities of Chinese herbal formula Yangyinjiedu (YYJD) and found that YYJD exhibits cytotoxicity against lung cancer cells. Transcriptome analysis indicated that 2178 genes were differentially expressed (*P* < 0.05) upon YYJD treatment, with 1464 being (67.2%) up‐regulated. Among these, we found that the tumour suppressor early growth response 1 (EGR1) is the most activated. We demonstrated that EGR1 contributes to YYJD‐induced apoptosis in A549. Through dissecting EGR1*‐*associated transcriptional network, we identified 275 genes as EGR1 direct targets, some targets are involved in apoptosis. Lastly, we observed that YYJD enhances EGR1 expression and induces cell death in tumour xenografts. Collectively, these findings suggest that YYJD exerts its anticancer activities through EGR1 activation, thus providing the evidence for its potential clinical application for lung cancer patients.

## 1 INTRODUCTION

Lung cancer is one of the most common cancer types that constitutes the leading cause of cancer‐related deaths worldwide.^1^ In 2015, the estimated numbers of new lung cancer cases and deaths in China were 4.292 million cases and 2.814 million respectively.^2^ The clinical interventions against lung cancer include surgery, chemotherapy, radiotherapy and target therapy. However, the 5‐year survival rate is lower than 20% in China.^3^ Due to the development of drug resistance^4^ and side effects,^5^ the effectiveness of these treatment options is limited, thus raising the need for alternative therapeutic approaches.

With a long history and extensive documentation of the clinical practices, traditional Chinese medicine (TCM) might represent a promising option.^6^, ^7^ Particularly, TCM has been widely used in clinical practice and shown significant anticancer effects.^8^, ^9^ Jinfukang (JFK), a Chinese herbal formula with 12 herbs, has been used to treat lung cancer in China.^5^, ^10^ Our previous studies indicated that it induces cellular apoptosis through activation of FAS and DR4, and exerts synergistic effects in combination with chemotherapy on lung cancer cell apoptosis.^11^, ^12^ Using computational algorithms, recently we optimized the ingredients of JFK formula and the optimized formula Yangyinjiedu (YYJD) exhibits anti‐tumour effect by inducing lung cancer cell senescence.^13^ In this study, we further examined the anti‐tumour mechanism of YYJD and demonstrated that YYJD induces apoptosis by activating transcriptional regulator EGR1 in lung cancer cells.

## 2 MATERIALS AND METHODS

### 2.1 Preparation of YYJD

The Formula of YYJD was prepared as previously described.^13^ *Astragalus*, *Radix Ophiopogonis*, *Paris polyphylla*, *Glossy Privet Fruit*, *Fiveleaf Gynostemma* were mixed and smashed according to the weight ratio of 3:1:2:1:1. Then, five times volumes of 70% alcohol and 30% pure water were added and extracted with ultrasonication for three times (60 minutes each time). The supernatant was collected, and the alcohol was removed through rotary evaporation, and then dried into powder by freeze drying. For in vitro experiments, the YYJD powder was dissolved in culture medium. The culture medium without YYJD was used as control.

### 2.2 Cell culture

A549 (TCHu150), NCI‐H2228 (SCSP‐5001), NCI‐H1299 (TCHu160), NCI‐H1975 (SCSP‐597), NCI‐HCC827 (TCHu153), mouse Lewis lung carcinoma (LLC, TCM 7) and human normal bronchial epithelial cells (16HBE) were obtained from the Shanghai Institute of Biochemistry and Cell Biology. Mycoplasma contamination was evaluated by PCR and all cell lines were found to be mycoplasma free. Cells were cultured in RPMI 1640 medium (Corning, USA) supplemented with 10% FBS (Gibco, USA) and 100 units per ml penicillin‐streptomycin solution at 37°C, 5% CO_2_ in a humidified incubator.

### 2.3 Cell viability analysis

Cells were seeded in 96‐well plates at a density of 5000 cells/well and cultured at 37°C, 5% CO_2_ in an incubator overnight, then treated with YYJD at different concentrations for 24, 48, and 72 hours respectively. At each time‐point, cell counting kit‐8 (CCK8, Sangon, China) was used to examine cell viability according to the manufacturer's protocol. The absorbance was measured at 450 nm through a spectrophotometric plate reader (Bio Tek, USA). Cell viability was calculated as described previously.^11^

### 2.4 Cell cycle analysis

Cells were seeded in 6‐well plates and treated with YYJD at different concentrations for 48 hours. All cells were collected and fixed with ice‐cold 75% ethanol at 4°C overnight. Cell cycle detection was performed according to our previous study.^13^

### 2.5 Cell apoptosis analysis

Cell apoptosis was detected by Annexin V‐FITC/PI Apoptosis kit (Sangon, China). Briefly, cells were seeded in 6‐well plates and treated with YYJD at different concentrations for 48 hours and harvested by trypsin (no EDTA), then washed twice with PBS and stained with Annexin V‐FITC/PI for 30 minutes. The cell apoptosis was detected by using BD LSRFortessa and analysed with FlowJo software.

### 2.6 Real‐time quantitative PCR

RNA extraction and reverse transcription were carried out according to our previous study.^14^ The mRNA levels of individual gene were detected by quantitative real‐time PCR (RT‐qPCR) using StepOne Plus Real‐Time PCR system. The primer sequences are shown in Table S1. The relative levels of mRNA were calculated as 2^ΔΔ^Ct.

### 2.7 RNA interference

Cells were seeded in 6‐well plates and transfected with *EGR1* and negative control siRNA using Lipofectamine 3000 (Invitrogen, USA) according to the manufacturer's instructions. The siRNA sequences are shown in Table S2. After 24 hours, when the transfection was done, the cells were treated with YYJD for 48 hours. Cell viability, apoptosis and mRNA expression were measured as described above.

### 2.8 Western bolt assay

Cells were lysed by RIPA buffer (Sangon, China) containing Proteinase inhibitor (Roche) and Pierce phosphatase inhibitor (Thermo Fisher, USA). Western blot was performed according to the standard methods as previous described.^15^ Briefly, equal amount of denatured protein from each sample was separated by 10% SDS–polyacrylamide gel and transferred to NC membranes. Primary antibodies against EGR1 (4154, Cell Signaling Technology, USA) was used for binding EGR1 protein, specifically, the primary antibody against GAPDH (2118, Cell Signaling Technology, USA) was used as an internal control. The protein was probed with goat anti‐rabbit IgG highly cross‐adsorbed secondary antibody (Invitrogen, USA) for 2 hours at room temperature.

### 2.9 Tumour growth assays

The logarithmic phase Lewis lung cancer cells at concentration of 1 × 10^6^ cells/mL, were inoculated in the right axillary subcutaneous inoculation, 0.2 mL per mouse. C57 BL/6 mice were weighed and randomly divided into four groups (n = 6), including control group (0.9% normal saline once a day for 14 days), treated with YYJD (18.8 g/kg), cisplatin (2 mg/kg, once every 4 days), YYJD (18.8 g/kg) combined with cisplatin (2 mg/kg, once every 4 days).^13^ Chinese herbs and saline were administered via gavage. Cisplatin was administered intraperitoneally (i.p.) with 200 μL. The control group and YYJD groups were administered every day. Tumour size was measured once every day and the volume was calculated as follows: volume = 0.5 length × width^2^.

### 2.10 Immunohistochemical analysis

The tumour tissues were fixed in 4% paraformaldehyde solution, embedded in paraffin permeabilized with 1% Triton‐X100 for 15 minutes, washed with PBS for three times. The tissues were first incubated with primary antibodies against EGR1 (4153, Cell Signaling Technology, USA), KLF11 (bs‐16096R, Bioss, China), and then incubated with a secondary antibody, according to the manufacturer's instructions.

### 2.11 RNA‐seq analysis

Total RNA of YYJD‐treated and untreated A549 cells was extracted using Trizol (Ambion, USA) according to the standard RNA isolation procedure. mRNA was purified using the NEBNext Poly (A) mRNA Magnetic Isolation Module (E7490, NEB, USA). Libraries were constructed using the NEBNext Ultra Directional RNA Library Prep Kit for Illumina (E7420, NEB, USA) and sequenced on NextSeq500 (Illumina, USA). The RNA‐seq raw data were mapped to the reference genome (hg19) using TopHat2.^16^ Cufflinks was used for characterizing the differential transcription pattern.^16^ The gene expression level was measured by fragments per kilobase of transcript per million fragments (FPKM). The genes were considered significantly differentially expressed between the untreated samples and YYJD‐treated samples when *P*‐value was less than 0.05 and |Log 2 Fold change| is larger than 2, The filtered differentially expressed genes (DEGs) were functionally annotated for GO analysis with DAVID (Database for Annotation, Visualization and Integrated Discovery).^17^ The raw data of RNA‐seq are available in the EMBL database (http://www.ebi.ac.uk/arrayexpress/) under accession number E‐MTAB‐7237.

### 2.12 ChIP‐seq analysis

ChIP‐seq libraries were prepared as previously described.^14^ Briefly, 5 million cells were cross‐linked by 1% formaldehyde (Sigma‐Aldrich, USA) for 10 minutes at room temperature and incubating with 125 mM glycine for 5 minutes to quench the cross‐linking reaction. Chromatin was sonicated into fragments with the size of 100 ~ 300 bp，and immunoprecipitated with protein A + G magnetic beads (Millipore, USA) coupled with 5 μg of anti‐EGR1 antibody (4154, Cell Signaling Technology) at 4°C overnight with rotation. After reverse cross‐linking, ChIP DNA and input DNA libraries were constructed by using the NEBNext Ultra II DNA Library Prep Kit for Illumina (E7645, NEB, USA) and sequenced on NextSeq 500 (Illumina, USA). The ChIP‐seq raw data were mapped to the reference genome (hg19) using Bowtie 2.^18^ We identified the enriched ChIP‐seq regions over background with the MACS2.^19^ The parameters of MACS were set as default except –nomodel = T, –shiftsize = 75, ‐q = 0.01. The raw data of ChIP‐seq are available in the EMBL database (http://www.ebi.ac.uk/arrayexpress/) under accession number E‐MTAB‐7236.

### 2.13 Statistical analysis

The data were presented as the mean ± standard deviation (SD). The differences between the groups were performed with one‐way ANOVA using GraphPad Prism 7.0 software. *P*‐values were calculated using two‐tailed Student's t‐test. All *P* values were adjusted with B‐H method.^20^ Values of *P* < 0.05, were considered to indicate a statistically significant difference.

## 3 RESULTS

### 3.1 YYJD exhibits cytotoxicity against lung cancer cell lines

**Figure 1:**
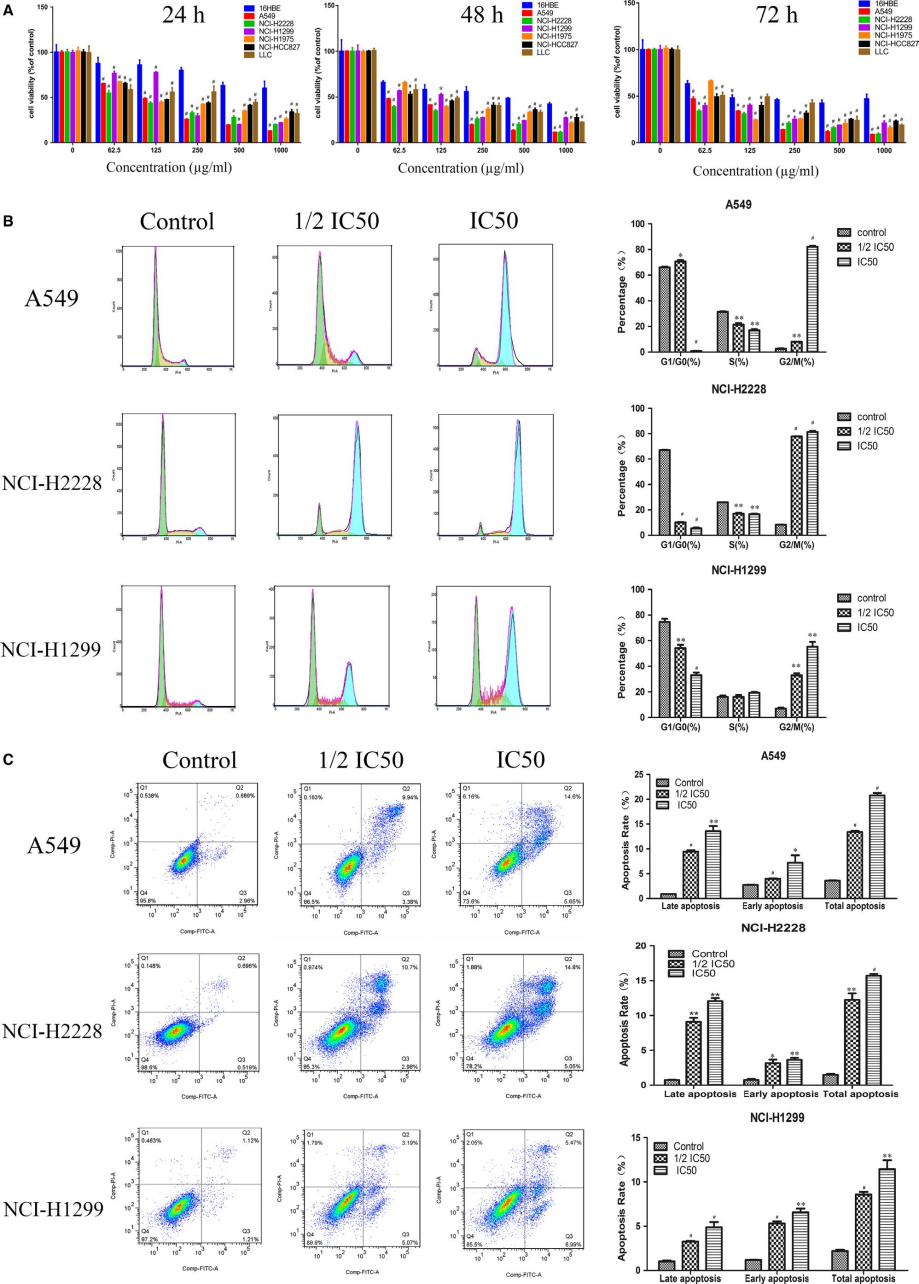
Yangyinjiedu (YYJD) induced anti‐tumour activities in lung cancer cells. A, The effects of various concentrations of YYJD on cellular proliferation of human lung cancer cell lines A549, NCI‐H2228, NCI‐H1299, NCI‐H1975, NCI‐HCC827, human normal bronchial epithelial cells (16HBE) and mouse Lewis lung carcinoma (LLC) were examined after 24, 48, and 72 h treatment respectively (*P < 0.05, ^#^ *P* < 0.001 compared with 16HBE group). B, YYJD induced cell cycle arrest at G_2_/M phase. C, YYJD enhanced apoptosis rates of lung cancer cells in a concentration‐dependent manner. Data are shown as Mean ± SD from at least three independent experiments (*P < 0.05, **P < 0.01, ^#^ *P* < 0.001 compared with control group)

We first examined whether YYJD exerts cytotoxicity on human lung cancer cell lines A549, NCI‐H2228, NCI‐H1299, NCI‐H1975, NCI‐HCC827, human normal bronchial epithelial cells (16HBE) and mouse Lewis lung carcinoma cells LLC. These cells were exposed to various concentrations of YYJD (0, 62.5, 125, 250, 500, and 1000 ug/mL) for 24, 48, and 72 hours respectively. The cell viability was determined by CCK8 assay. As shown in Figure 1A, cell viability was decreased in these tested lung cancer cell lines in a dose‐ and time‐dependent manner when compared with 16HBE (*P＜0.05, ^#^ *P*＜0.001). Moreover, the viable cell number was reduced with the YYJD treatment, demonstrated by the half maximal inhibitory concentration (IC_50_) (Table S3).

We next characterized the cell cycle‐related events by flow cytometry with the cells treated by YYJD for 48 hours. Consistent to our previous observation, we found that the treatment of A549, NCI‐H2228 and NCI‐H1299 cells with YYJD resulted in a significant increase in the proportion of cells at the G2/M phase and a reduction in the proportion of cells at the G_1_/G_0_ phase (Figure 1B).

To ask whether YYJD inhibits cell viability by inducing apoptosis, the morphological changes in A549, NCI‐H2228 and NCI‐H1299 cells treated with YYJD for 48 hours were evaluated under fluorescence microscope or phase‐contrast microscopy. We observed that YYJD treatment triggered lung cancer cells exhibiting apoptotic morphology with condensed nuclei, membrane blebbing, vacuolation in the cytoplasm and formation of apoptotic bodies (Figure S1).

To further analyse the apoptotic features of YYJD‐treated lung cancer cells, we performed Annexin V‐FITC/PI double staining assay. Compared with the control, the total apoptotic cells were significantly increased (*P < 0.05, **P < 0.01, ***P < 0.001) in dose‐dependent manner upon YYJD treatment for 48 hours in A549, NCI‐H2228 and NCI‐H1299 (Figure 1C). In particular, we found that the late apoptotic cells were much more than the early ones in A549 and NCI‐H2228, while such phenomenon is absent in NCI‐H1299, suggesting that A549 and NCI‐H2228 are more sensitive to YYJD. The total apoptotic rate of YYJD‐treated A549 is the highest among the lung cancer cell lines examined, A549 was thus used for further characterization.

### 3.2 YYJD induced transcriptome analysis in lung cancer cells

**Figure 2:**
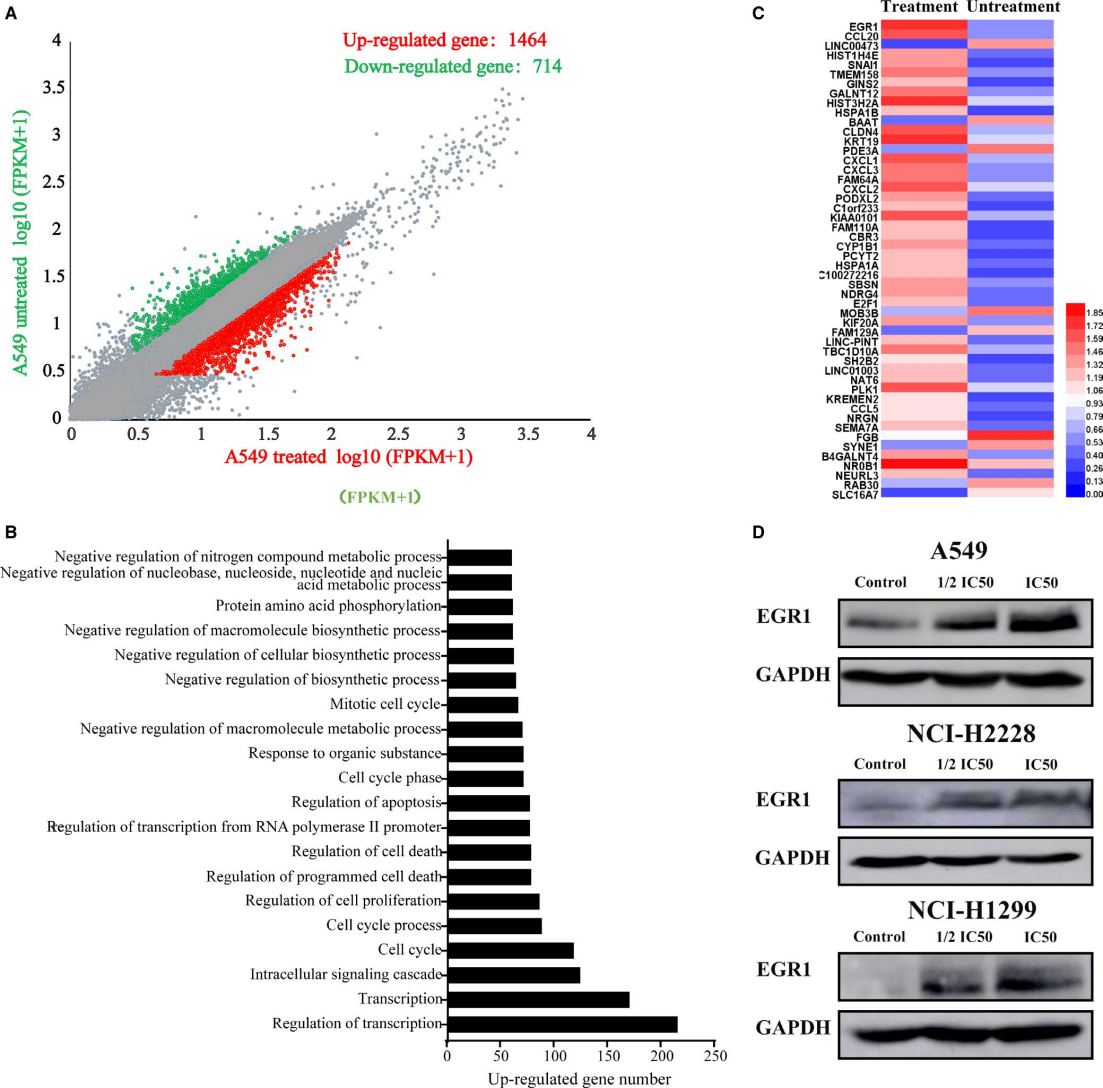
Characterization of the Yangyinjiedu (YYJD) induced differential gene expression in lung cancer cells. A, Scatter plot showed the differential gene expression pattern in A549 with YYJD treatment or with no YYJD treatment. Expression was shown as log10 of the FPKM+1, including up‐regulated (red) and down‐regulated (green) genes. B, Gene ontology analysis of the significantly differentially expressed genes in YYJD‐treated A549. C, Heatmap showed the 50 most up‐regulated and down‐regulated in YYJD‐treated A549. D, The alteration in early growth response 1 protein levels in YYJD‐treated lung cancer cell lines was examined by Western blot

To understand the underlying molecular mechanisms of the growth inhibition and apoptosis effects induced by YYJD, we next performed a transcriptome analysis to investigate differential gene expression in YYJD‐treated A549 through RNA‐seq. In total, 47.1 million and 93.8 million reads were generated from two YYJD‐treated replicates and 52.2 million reads for untreated A549 cells, and 88.3%, 57.1% and 91.8% of which were uniquely mapped to the human genome (hg19), corresponding to 23 453 and 19 009 expressed genes respectively (Table S4). Compared with the control, 2178 differentially expressed genes (*P* < 0.05) were identified, with 1464 (67.2%) up‐regulated and 714 (32.8%) down‐regulated (Figure 2A and Table S5). To verify whether such expression pattern is present in other lung cancer cells, we randomly chose nine apoptosis‐ or cell cycle‐related genes and performed RT‐qPCR assay with NCI‐H1299. We found that these genes show similar patterns to what we observed in A549 (Figure S2).

Using DAVID, we next performed gene ontology (GO) analysis with differentially expressed genes upon YYJD treatment.^17^ The top 20 GO terms are shown in Figure 2B, including cell proliferation‐ and cell death‐related processes. In particular, we did observe that apoptosis‐related process is significantly enriched among these GO terms (Figure 2B).

### 3.3 *EGR1* is involved in YYJD‐induced apoptosis in lung cancer cells

The top 50 most differentially expressed genes are shown in Figure 2C. Among them, *EGR1* was most up‐regulated. The protein encoded by *EGR1* is a nuclear protein and functions as a transcriptional regulator. Moreover, previous studies demonstrated it is involved in apoptosis.^21^, ^22^, ^23^, ^24^, ^25^, ^26^ We observed that YYJD treatment induced up‐regulation of EGR1 at protein level (Figure 2D). Such YYJD‐induced EGR1 activation was also observed in lung cancer cell lines NCI‐H2228 and NCI‐H1299 (Figure 2D). These observations suggest EGR1 potentially contributes to apoptosis in YYJD‐treated lung cancer cells.

**Figure 3:**
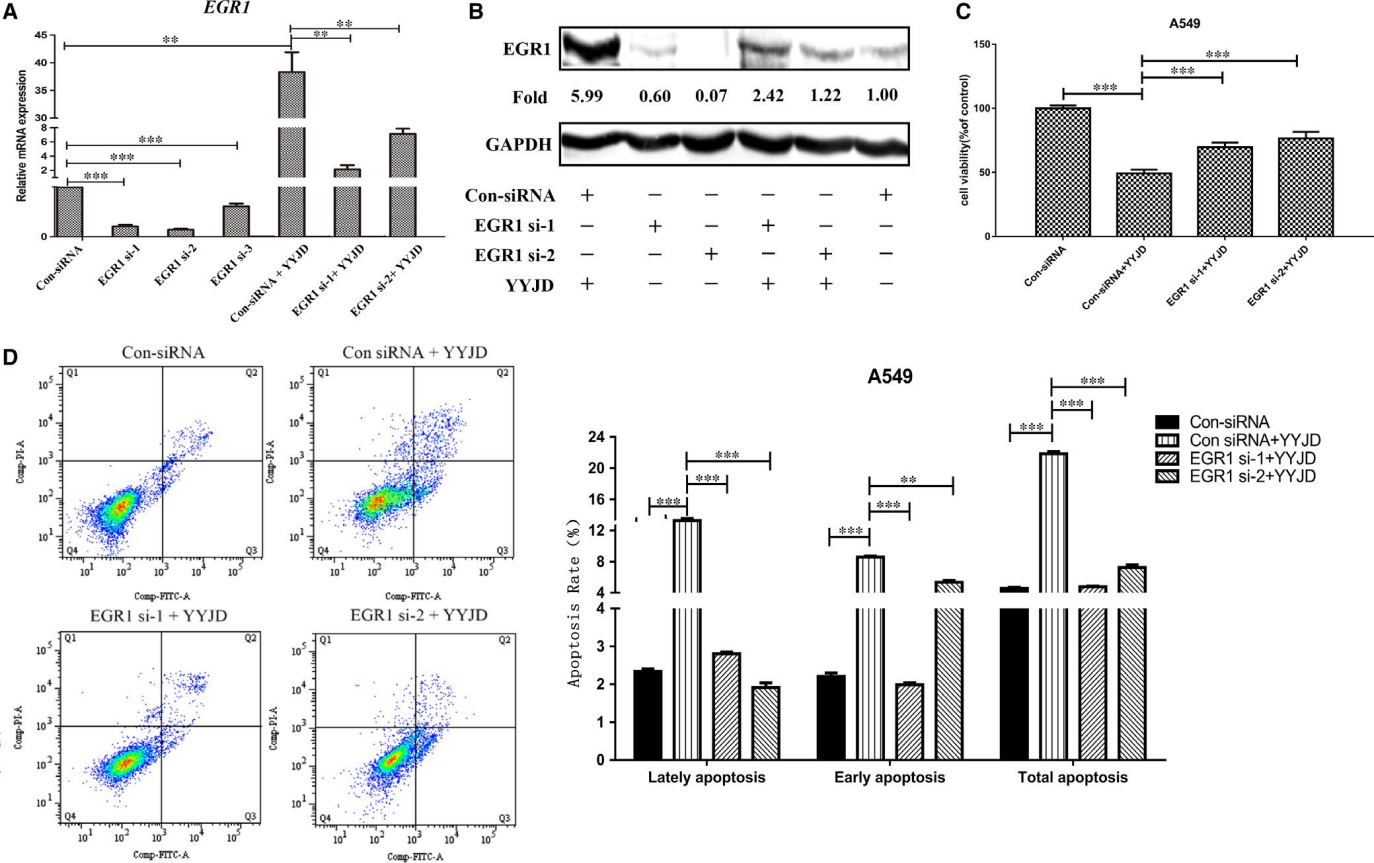
Knockdown of early growth response 1 (EGR1) attenuated the Yangyinjiedu induced pro‐apoptosis effect in A549. A, The expression levels of *EGR1* mRNA were detected by quantitative real‐time PCR. B, The expression levels of EGR1 protein were measured by Western blot. C, Cell viabilities were examined by CCK8 assay. D, Apoptotic cells were measured by flow cytometric assay. Ratios of early apoptosis and total apoptosis were analysed with flowjo software (**P < 0.01, ***P < 0.001 compared with control group)

To further verify the functional relevance of *EGR1* activation in YYJD‐induced apoptosis, we performed siRNA assay to suppress *EGR1* expression. siEGR1 knockdown cells were treated with YYJD (63 ug/mL) for 48 hours. Although EGR1 is activated upon YYJD treatment, it is remarkably suppressed by siRNA both in mRNA and protein levels (Figure 3A, B). Moreover, we observed a considerable decrease in both viability inhibition and pro‐apoptosis activity in YYJD‐treated A549 cells when *EGR1* is suppressed (Figure 3C, D). These results suggest that EGR1 is involved, in part at least, in YYJD‐induced apoptosis in lung cancer cells.

### 3.4 Transcriptional network mediated by *EGR1* in YYJD‐treated lung cancer cells

**Figure 4:**
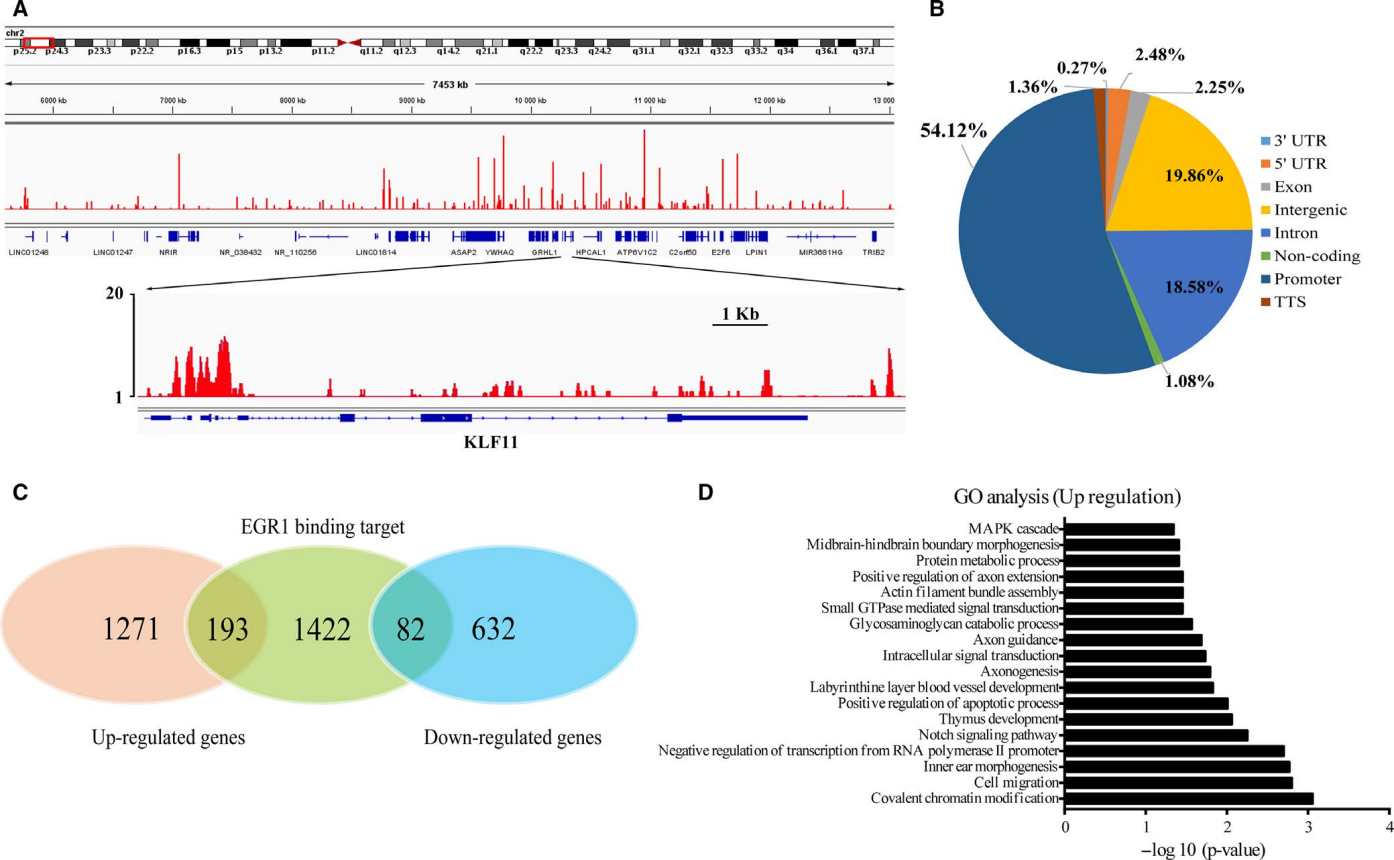
Characterization of early growth response 1 (EGR1) directed transcriptional network in A549. A, A snapshot of the IGV genome browser showed the sequencing read signals of EGR1 binding sites. B, Genomic distribution of EGR1 binding sites across the Yangyinjiedu (YYJD) treated A549 genome. C, Venn diagram of differentially expressed genes and EGR1‐bound genes in YYJD‐treated A549. D, Gene ontology analysis of the up‐regulated EGR1 target genes (*P* < 0.05)

The nuclear protein EGR1 functions as a transcription factor.^27^ Given the pro‐apoptosis activity induced by EGR1 activation (Figure 3D), we next sought to understand how EGR1 exerts its pro‐apoptosis activity through modulating its downstream target genes. To this end, we performed chromatin immunoprecipitation (ChIP) coupled with deep sequencing (ChIP‐seq) analysis to interrogate EGR1 binding targets across the whole genome in YYJD‐treated A549 cells (Figure 4A). Totally, we generated 97.7 million reads, which yielded 4892 binding sites (*P* < 0.01) (Table S6). To identify EGR1‐bound target genes, we next examined the genomic distribution of these binding loci in relation to the nearest transcript unit. Similar to what we observed previously,^28^ we found that the binding sites of EGR1 are located in distinct regions, including promoter, exon, intron or intergenic regions (Figure 4B). We generated a set of genes that contain EGR1 binding sites in the vicinity of −5 ~ +2 kb of transcript start sites, yielding 1697 EGR1‐bound genes.

To understand how EGR1 affects the expression status of its downstream target genes in YYJD‐treated lung cancer cells, we analysed the EGR1‐associated transcription network by intersecting ChIP‐seq data with the transcriptome data. As mentioned above, we found that YYJD induced 2178 genes to be expressed differentially (*P* < 0.05). Among these genes, 275 genes are bound by EGR1, with 193 up‐regulated and 82 down‐regulated (Figure 4C) (Table S7). Thus, these EGR1‐bound and YYJD‐responsive genes are potentially involved in biological activities we observed in YYJD‐treated lung cancer cells. To verify this assumption, we performed GO analysis of EGR1‐bound and up‐regulated genes with DAVID.^17^ We did find that some up‐regulated genes were enriched in GO terms positive regulation of apoptotic process (Figure 4D), including *ABR, ING2, CYP1B1, HIP1R, KLF11, VAV2, TGFB1, BCL2L11,* and *DUSP6.* These results suggest that EGR1 activates the apoptosis‐related genes in YYJD‐treated lung cancer cells.

### 3.5 YYJD enhances EGR1 expression and induces cell death in tumour xenografts

**Figure 5:**
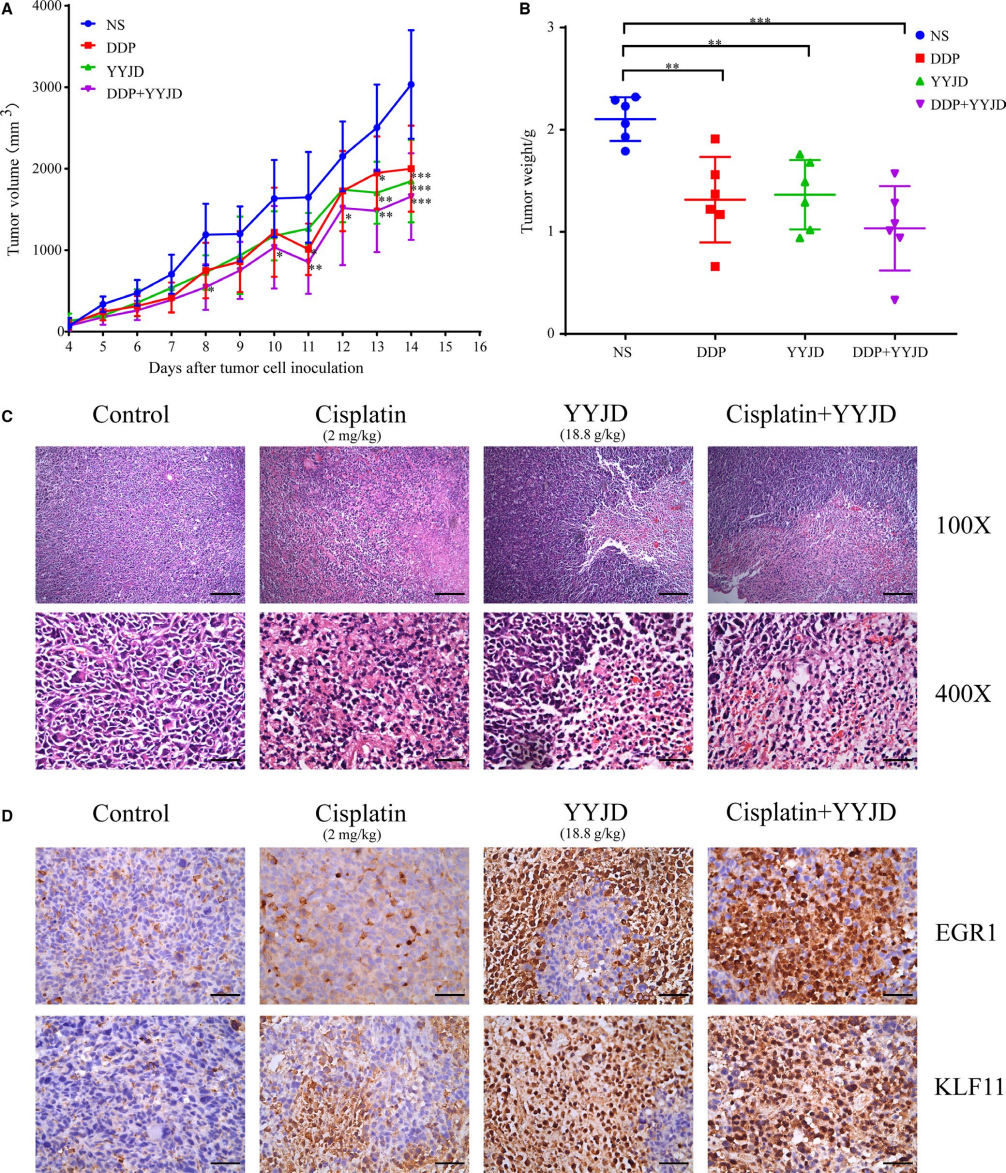
Tumour inhibitory effect of Yangyinjiedu in vivo. A, The tumour volumes were measured once every day. *P < 0.05, **P < 0.01 and ***P < 0.001. B, The comparison of tumour weights of four groups. *P < 0.05, **P < 0.01 and ***P < 0.001. C, Haematoxylin eosin staining of the tumour tissues (100× and 400× magnification). Representative images were shown from six mice in each group. D, The expression of early growth response 1 and KLF11 in tumour xenograft tissues was detected by immunohistochemistry (400×). Scale bars: 200 μm

Given the observation that YYJD exerts the anti‐tumour effect through EGR1 activation in vitro, its anti‐tumour activity in vivo remains unclear. Thus, we generated lung cancer cell tumour xenografts, which were subsequently treated with YYJD, cisplatin, respectively, or in combination. We found that both YYJD and cisplatin inhibit the growth of tumour xenografts, demonstrated by the significant decrease in both tumour volume and tumour weight; and this effect looks more obvious when they were applied in combination (Figure 5A, B).

We next performed immunohistochemical analysis and found that YYJD induces cell death in tumour xenografts (Figure 5C). Importantly, we observed the obvious increase in protein level of EGR1 (Figure 5D). In addition, we found an apoptosis‐related gene *KLF11*,^29^, ^30^, ^31^ one of the EGR1 downstream targets identified in this study (Figure 4A), is also activated upon YYJD treatment (Figure 5D).

## 4 DISCUSSION

It is documented that TCM has been widely used for thousands of years in China. Recently, a large number of studies have suggested that TCM is useful in the treatment of various types of cancers.^8^ In our previous studies, we reported that TCM Jingfukang and its derivative YYJD exert the anti‐tumour effect by inducing apoptosis and senescence in lung cancer cells.^11^, ^13^ Moreover, we observed that such anti‐lung cancer activity involves alteration of histone modification in lung cancer cells.^32^

Consistent to our previous observations, in this study we found that YYJD inhibits cell proliferation and induces cell cycle arrest and apoptosis in lung cancer cells (Figure 1A‐C). The YYJD‐induced cell cytotoxicity seems to be cancer cell‐specific, as less cytotoxicity was observed in YYJD‐treated normal lung epithelial cells (Figure 1A). Through transcriptome analysis in YYJD‐treated A549 we found that YYJD induces 2178 genes to be differentially expressed, with the majority being up‐regulated (Figure 2A).

Among the YYJD‐responsive genes, *EGR1* raised our attention due to its highest transcriptional alteration (Figure 2C) and involvement against lung cancer.^33^, ^34^, ^35^ EGR‐1 is a transcription factor of zinc finger family. It can be induced by a wide range of extracellular stimuli (including growth factors and cytokines) and activates the downstream target genes through the consensus GC‐rich sequence 5’‐ MCGCCCACDC‐3’0.^36^ Recently, it has been reported that the compound ciglitazone induces EGR‐1 and leads to inhibition of lung cancer cell proliferation.^33^ In this study, we observed that TCM YYJD induces proliferation inhibition and apoptosis in lung cancer cells (Figure 1A). We then demonstrated that the YYJD‐induced pro‐apoptosis activity is mediated, at least partly, by activating EGR1 (Figure 2C, D and Figure 3). We further delineate the EGR1 target genes in YYJD‐treated A549, including some apoptosis‐related genes such as *KLF11,* 29, 30, 31 *BCL2L11* 37, 38, 39, and *DUSP6.* 40, 41 These observations suggest that the apoptosis of lung cancer cells induced by YYJD is possibly mediated by EGR1‐bound target genes.

In brief, we demonstrated that the transcription factor EGR1 is activated by TCM YYJD and such activation is involved in YYJD‐induced apoptosis in lung cancer cells. Thus, our study provided a novel insight to understand the anti‐tumour mechanism of Chinese herb YYJD.

## CONFLICT OF INTEREST

The authors declare that they have no competing interests.

## AUTHOR CONTRIBUTIONS

X. Zhao. and L. Xu. designed the research. W. Yang, L. Bi., Y. Gu., J. Lu. and J. Yao. performed the experiments. Y. Kang., Q. Zhao., L. Jiao., D Zhou, J Sun, X. Zhao. and L. Xu. analysed the data. W. Yang. and X. Zhao. wrote the manuscript. X. Zhao. and L. Xu. edited the manuscript.
