# Supplementary material for: Herbal formula Yangyinjiedu induces lung cancer cell apoptosis via activation of early growth response 1
Source: J Cell Mol Med. 2019 Jun 25;23(9):6193–202. doi: 10.1111/jcmm.14501 (PMC6714142; doi:10.1111/jcmm.14501)
Supplement: Supplementary file 4 [file JCMM-23-6193-s004.docx]

**Table S1.** List of qPCR primers used for mRNA expression

| Primer name | Sequence |
| --- | --- |
| *EGR1* forward primer | TGACCGCAGAGTCTTTTCCT |
| *EGR1* reverse primer | TGGGTTGGTCATGCTCACTA |
| *GAPDH* forward primer | ACAACTTTGGTATCGTGGAAGG |
| *GAPDH* reverse primer | GCCATCACGCCACAGTTTC |
| *TM4SF4* forward primer | CGTAGGAGGAATCCAGATGGT |
| *TM4SF4* reverse primer | CTCGGAGGTTTAAACGGGTC |
| *CDK6* forward primer | ACAGAGCACCCGAAGTCTTG |
| *CDK6* reverse primer | TCAACATCTGAACTTCCACGAA |
| *APOL1* forward primer | CTGCTACTCCTGCTGACTGA |
| *APOL1* reverse primer | TCATCTGCCTCATTCCTGGG |
| *TGFB1* forward primer | GCTAATGGTGGAAACCCACAAC |
| *TGFB1* reverse primer | GAGCAACACGGGTTCAGGTA |
| *IGFBP6* forward primer | CGAGGGGCTCAAACACTCTA |
| *IGFBP6* reverse primer | CATCCGATCCACACACCAG |
| *NFKBIA* forward primer | AGCTCCGAGACTTTCGAGGA |
| *NFKBIA* reverse primer | CACCAAAAGCTCCACGATGC |
| *SFRP1* forward primer | GAGTTTGCACTGAGGATGAAAA |
| *SFRP1* forward primer | GCTTCTTCTTCTTGGGGACA |
| *BCL2A1* forward primer | AGGTGTGTGATTGTGCCATT |
| *BCL2A1* reverse primer | AATTGCCCCGGATGTGGATA |

**Table S2.** List of siRNA sequences used in *EGR1* knockdown experiment.

| Primer name | Sequence |
| --- | --- |
| siEGR1-1 | CCAUGGACAACUACCCUAATT |
|  | UUAGGGUAGUUGUCCAUGGTT |
| siEGR1-2 | GCCUAGUGAGCAUGACCAATT |
|  | UUGGUCAUGCUCACUAGGCTT |
| siEGR1-3 | GCAAGAGGCAUACCAAGAUTT |
|  | AUCUUGGUAUGCCUCUUGCTT |
| Negative control | UUCUCCGAACGUGUCACGUTT |
|  | ACGUGACACGUUCGGAGAATT |

**Table S3.** IC_50_ values for the normal lung cell line and lung cancer cell lines of YYJD treated.

| Cell lines | IC_50_ in ug/ml | | |
| --- | --- | --- | --- |
|  | 24h | 48h | 72h |
| 16HBE | 1639 | 392.5 | 253.5 |
| A549 | 112.5 | 62.77 | 57.3 |
| NCI-H2228 | 115 | 55.94 | 40.91 |
| NCI-H1299 | 194.7 | 105.4 | 53.2 |
| NCI-H1975 | 152.7 | 118.9 | 85.33 |
| NCI-HCC827 | 188.1 | 116.6 | 74.77 |
| LLC | 247.8 | 127.7 | 102 |

**Table S4.** The statistics of RNA‑seq data sets

| Name | Total reads | Mapped reads | Unique mapped | mapped rates |
| --- | --- | --- | --- | --- |
| Control | 52215429 | 49392598 | 47956575 | 94.6% |
| Treatment-1 | 47069716 | 43157439 | 41571861 | 91.7% |
| Treatment-2 | 93871560 | 55690310 | 53625218 | 59.3% |


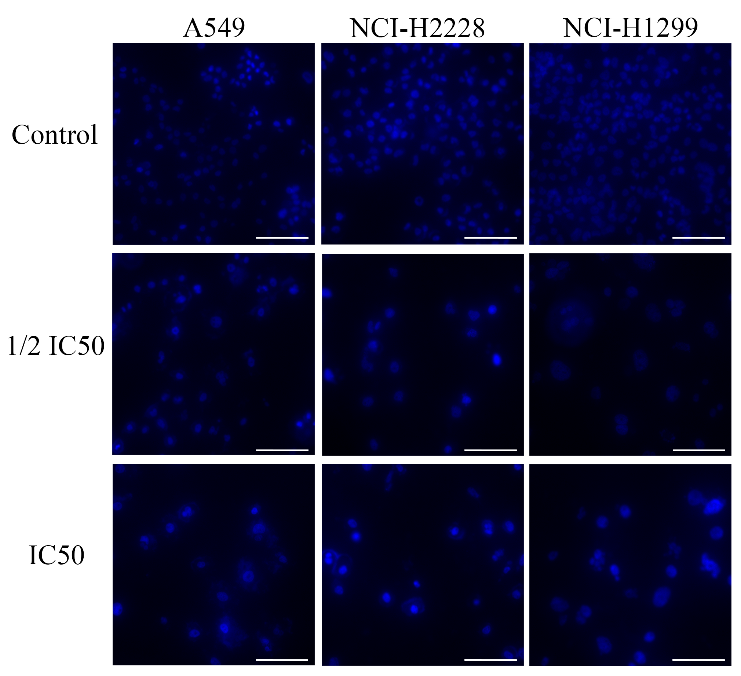


**Figure S1.** The effects of apoptosis induced by YYJD. Hochest 33342 staining of A549, NCI-H2228 and NCI-H1299 cell lines with different concentrations of YYJD for 48 hours. (Scale bars: 100 μm)


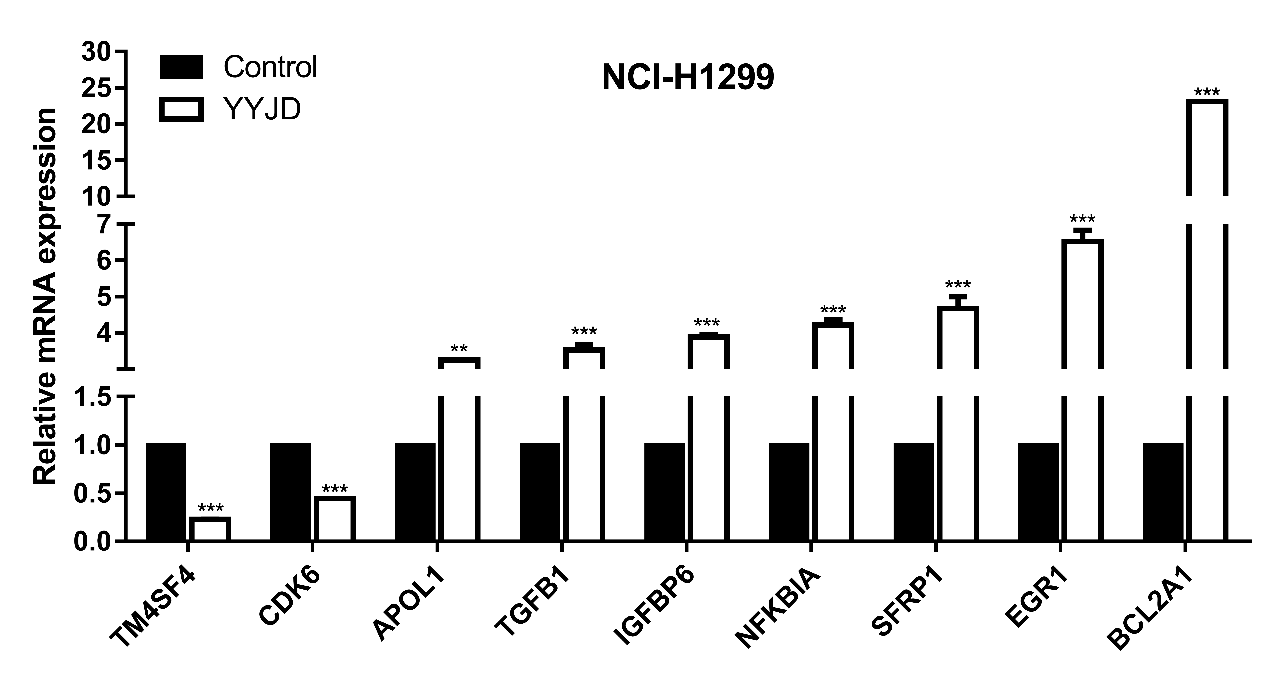


**Figure S2.** Expression levels of genes were examined by qPCR. NCI-H1299 cells were treated with YYJD (105 μg/ml) for 48 hours. Expression levels of *TM4SF4, CDK6, APOL1, TGFB1, IGFBP6, NFKBIA, SFRP1, EGR1* and *BCL2A1* were examined by RT-qPCR.
